# Mitochondrial potassium channels: mitochondria-specific mechanism of regulation

**DOI:** 10.1007/s12551-025-01385-9

**Published:** 2025-11-27

**Authors:** Joanna Lewandowska, Barbara Kalenik, Antoni Wrzosek, Bogusz Kulawiak, Piotr Bednarczyk, Barbara Zablocka, Adam Szewczyk

**Affiliations:** 1 Laboratory of Intracellular Ion Channels, Nencki Institute of Experimental Biology, Polish Academy of Sciences, Warsaw, Poland; 2 Department of Physics and Biophysics, Institute of Biology, Warsaw University of Life Sciences – SGGW, Warsaw, Poland; 3 Mossakowski Medical Research Institute, Polish Academy of Sciences, Warsaw, Poland

**Keywords:** Mitochondria, Mitochondrial potassium channels, Respiratory chain, Reactive oxygen species, Kinases, Metabolism, Potassium channels openers

## Abstract

Potassium channels identified in the plasma membrane play a crucial role, particularly in generating action potentials in excitable cells. Recently, potassium channels have also been discovered in intracellular organelles, including the inner mitochondrial membrane (IMM), which share many properties with their plasma membrane counterparts. Mitochondrial potassium channels exhibit similar biophysical, pharmacological, and regulatory characteristics, reflecting their common molecular origin. However, differences in potassium channel activity may result from differences in isoforms as well as from the specific ionic, protein, and lipid environments associated with their distinct subcellular locations. In particular, the IMM imposes unique conditions that shape the regulation of mitochondrial potassium channels. These include close proximity to the respiratory chain, high mitochondrial metabolic activity, a pronounced transmembrane potential, and pH gradients. This review examines how these mitochondrial-specific factors influence the function of mitochondrial potassium channels. A deeper understanding of how the IMM environment modulates mitochondrial channel activity will not only expand our knowledge of mitochondrial physiology but may also pave the way for new therapeutic strategies targeting mitochondrial dysfunction and the role of mitochondrial potassium channels in human diseases.

## Introduction

 Following the identification and study of channels found in cell membranes, significant progress has been made over the past decades in the identification and characterization of intracellular ion channels in a wide range of organisms (Hu et al. 2024). These channels are found in majority of subcellular membranes, including mitochondria, the endoplasmic/sarcoplasmic reticulum, lysosomes, peroxisomes, the nucleus, and in chloroplasts. Each class of intracellular ion channel exhibits specific ion selectivity, distinct gating mechanisms, defined regulatory interactions, and a pharmacological profile (Kaczmarek and Jonas 2004). These channels play crucial roles in maintaining organelle ions homeostasis, coordinating calcium ions fluxes and redox signaling, regulating energy metabolism, and intracellular membranes potential. Consequently, intracellular ion channels have appeared as a rapidly expanding field of research, with growing relevance for understanding cellular physiology as well as for the development of targeted therapeutic interventions (Zifarelli et al. 2024).

Among the various intracellular organelles, mitochondria host a particularly diverse set of ion-conducting pathways, which are recognized as critical regulators of cellular bioenergetics (Szabo and Szewczyk 2023). Mitochondrial ion channels contribute to the control of mitochondrial membrane potential, ATP synthesis efficiency, Ca^2+^ handling, redox balance, and reactive oxygen species (ROS) synthesis (O’Rourke 2007; Lewandowska et al. 2024). These channels are located either in the outer mitochondrial membrane (OMM) or the inner mitochondrial membrane (IMM), the latter being especially important due to its involvement in oxidative phosphorylation (O’Rourke et al. 2005). Within the IMM, potassium channels (mitoK channels) have attracted considerable attention, as they are implicated in the modulation of mitochondrial volume, respiratory chain activity, and cytoprotective responses, particularly under conditions of ischemia–reperfusion injury (Urbani et al. 2021). Moreover, mitoK channels have been shown to play a pivotal role in regulating the metabolism of cancer cells and may constitute a critical point in the induction of cell-death pathways across several tumor types (Checchetto et al. 2021). Despite significant advances in identifying putative molecular candidates, the precise molecular identity, regulatory mechanisms, and physiological functions of several mitoK channels remain subjects of ongoing investigation. Consequently, mitoK channels represent a dynamic and evolving area of research at the intersection of membrane biophysics and mitochondrial physiology (Szewczyk et al. 2025).

The concept of K^+^ transport across the IMM emerged in the late 1970 s, when early swelling experiments demonstrated that mitochondria are capable of accumulating K⁺ in a membrane potential-dependent manner. Initial studies suggested that K⁺ fluxes could modulate mitochondrial matrix volume and thereby influence oxidative phosphorylation efficiency (Szabo and Zoratti 2014). However, the discovery of ATP-sensitive potassium (mitoK_ATP_) channel-like activity in mitochondria in the early 1990 s provided the first strong evidence for the existence of discrete K⁺-selective conductive pathways within mitochondria (Inoue et al. 1991). Subsequent advances in patch-clamp techniques adapted to mitoplasts (inner mitochondrial membrane) enabled more direct electrophysiological characterization of several distinct potassium channels in the IMM, including large-conductance Ca^2^⁺-activated (mitoBK_Ca_) channels and acid-sensitive K⁺ channels (e.g. TASK-3). Over time, pharmacological studies, combined with genetic and proteomic approaches, have expanded the catalog of putative mitochondrial K⁺ channel candidates and linked their activity to cellular protective mechanisms, particularly in the context of ischemic preconditioning (Checchetto et al. 2021). Despite these advances, the molecular identity and regulation of several mitoK channels continue to be debated due to the highly specialized and compartmentalized environment of the IMM.

The regulatory mechanisms controlling mitoK channels differ in some respects from those operating in their plasma membrane counterparts, largely due to the unique biochemical and biophysical microenvironment of the mitochondrial matrix and inner mitochondrial membrane. On closer examination, several mitochondrial K⁺ (mitoK) channels share structural or pharmacological features with well-characterized plasma-membrane channels (e.g. ATP-sensitive, Ca^2^⁺-activated, or pH-sensitive K⁺ channels) (Szabo and Szewczyk 2023). However, their activity in mitochondria is determined by local factors, notably the highly negative membrane potential, ions-gradient, redox conditions, and interactions with respiratory-chain complexes. Matrix ATP/ADP ratio, Ca^2^⁺ concentration, and pH differ markedly from cytosolic values and can exert specific modulatory effects on mitoK channel gating (Kulawiak et al. 2021). Additionally, the IMM is enriched in cardiolipin and exhibits a different lipid composition compared to the plasma membrane, which may affect channel conformation, biophysical, and pharmacological properties. MitoK channels are also positioned within a tightly regulated metabolic network, often functionally coupled to redox signaling pathways. Consequently, even channels with analogous structural motifs to those in the plasma membrane may display divergent regulatory responses once embedded in the mitochondrial environment. Understanding how the unique mitochondrial environment alters mitoK channel function is thus essential for understanding their physiological roles and potential therapeutic value (Kampa et al. 2022).

In this article, we review the specific regulatory mechanism of the mitoK channels with respect to their localization in the inner mitochondrial membrane and discuss the implications of these regulations for cellular functions. This article expands upon concepts introduced in previous manuscript (Szewczyk 2024), providing a mechanistic analysis of mitochondrial specific regulatory processes affecting mitoK channels.

## Redox regulation of mitochondrial potassium channels

Mitochondria are semi-autonomous organelles in which a significant amount of reactive oxygen (ROS) and nitrogen (RNS) species are synthesized (Hernansanz-Agustín and Enríquez 2021; Murphy 2009). ROS and RNS synthesized in cells are regulated by antioxidant enzymes, such as catalase and specific members of the peroxiredoxin family, superoxide dismutase, and glutathione peroxidase, which convert them into harmless compounds, thereby reducing but not completely preventing effects of ROS and RNS (Balaban et al. 2005). Similar to a number of proteins occurring in the cell, mitoK channels are subject to redox processes, which, depending on the intensity, can be a part of the homeostatic signaling pathway or lead to apoptosis or necrosis (Tretter et al. 2021). Pathological synthesis of ROS in mitochondria increases the probability of redox modification of mitoK channels, which consequently influences changes in channel activity (Lewandowska et al. 2024; Balaban et al. 2005). Several studies have shown regulation of mitoK_ATP_ activity by redox state (Facundo et al. 2007; Queliconi et al. 2011; Perrelli et al. 2013). Although preconditioning against brain and cardiac ischemia/reperfusion injury by mitoK channels openers is well described (see below), ROS scavenger, 2-mercaptopropionylglycine (MPG), partially reduced the protective effect of catestatin. Furthermore, mitochondrial ROS generation has been correlated to the increased K^+^ flux into the mitochondrial matrix (Aggarwal and Makielski 2013).

It is also important to underline that mitoK channels’ activity also influences the level of ROS synthesis (Akopova and Smirnov 2025; Liang et al. 2025; Di Marco et al. 2024; Gómez Del Val et al. 2024). The mitoK channels play an important role in modulating the transmembrane potential (ΔΨm) of the IMM, which significantly affects ROS synthesis (Lewandowska et al. 2024; Berry et al. 2018). For instance, the presence of a pore-forming subunit of the mitoBK_Ca_ channel decreases the levels of ROS in mitochondria (Kulawiak et al. 2023). Moreover, changing mitoK channels’ activity can reduce hydrogen peroxide synthesis in isolated rat brain mitochondria, as demonstrated for mitoBK_Ca_ channels using synthetic activators of this channel, CGS 7184 and NS 1619 (Kulawiak et al. 2008). On the contrary, increased superoxide generation from complex I of the electron transport chain (ETC) was reported after opening mitoK_ATP_ channel in cardiomyocytes (Andrukhiv et al. 2006). This effect can be related to usual inhibition of the activity of this channel by a large amount of ATP generated by cardiomyocyte mitochondria (Ding et al. 2023).

The mitoK channels’ effect on ROS synthesis can be used in cancer therapy. Second near-infrared (NIR-II) responsive conjugated oligomer nanoparticles can disrupt the redox balance by releasing a specific inhibitor of the potassium channel via a temperature-sensitive liposome, thus altering the redox balance of mitochondria (Li et al. 2023). Moreover, a recent study investigating mitoK channels as regulators of ROS presented highly promising results (Russo et al. 2024). Authors of this study engineered from TASK1 channel a synthetic potassium channel that is sensitive to supraphysiological ROS levels and expressed in mitochondria. Expression of this channel significantly reduced elevated ROS levels in myoblasts from a patient affected by Leigh syndrome, restoring levels to those observed in healthy controls. Importantly, there was no obvious effect of this channel in healthy cells.

Damaging processes occurring in cardiac cells resulting from ischemia/reperfusion (I/R) can also be alleviated by the use of mitoK channel openers (O'Rourke 2004; Xu et al. 2002). Thus, opening of mitoK channels used as a preconditioning procedure decreases ROS synthesis and tissue damage (Roslan et al. 2025; Szteyn and Singh 2020; O'Rourke 2004). Moreover, activation of mitoK channels at the onset of reoxygenation also protects the heart from I/R injury probably by reducing excess generation of ROS (Jin et al. 2012).

Unraveling the complex network of functional relationships in cells is an extremely difficult task; however, acrolein (reactive aldehyde) treatment of HPAEC cells increased both mitochondrial ROS and amount of Kv1.5 mRNA and protein (Ouyang et al. 2012). To sum up, mitoK_ATP_ channels are known to regulate and to be regulated by mitochondrial redox state (Pereira et al. 2013).

In summary, mitoK channels and ROS engage in a distinctive form of cross-talk. First, mitoK channels are subject to regulation due to their close proximity to the ROS synthesis. Second, activation of these channels influences ROS synthesis by dissipating the mitochondrial membrane potential. This bidirectional interaction—where channels are both regulated by and regulate ROS level—is a unique feature of mitoK channels.

## Electron transport chain and potassium channel regulation

The regulation of mitoK channels by the electron transport chain (ETC) is a complex and important topic in cellular bioenergetics. The ETC involves complex redox reactions that generate a proton motive force, which is then utilized for ATP synthesis in mitochondria. Some respiratory redox centers, including complexes I and III, are known to produce ROS (Palma et al. 2024). As mentioned in the previous chapter, these mitochondrial ROS can impact the activity of mitoK channels. Also, it has been described that the presence of these channels is important for the ROS regulation levels in mitochondria (Kulawiak et al. 2023). However, there are emerging indications that suggest a more direct mechanism by which the ETC might regulate mitoK channels’ activity by putative functional and structural coupling.

Interactions of mitoK channels with various mitochondrial proteins, several of which constitute ETC complexes, are well documented (Lewandowska et al. 2024). For example, it has been suggested that mitoK_ATP_ channels may interact with succinate dehydrogenase (Ardehali et al. 2004). Also, it has been shown that mitoBK_Ca_-β1 may be involved in oxygen metabolism, proton transfer, and cytochrome *c* release in cardiac ventricular mitochondria. It is suggested that molecular mechanism is based on direct interactions of α subunits of the mitoBK_Ca_ channel and mitochondrial cytochrome c oxidase (Ohya et al. 2005). Furthermore, a recent study provides new insights into the mechanism of action of psoralen (a bioactive natural compound) derivatives acting on mitoKv1.3, revealing the association of the mitochondrial channel with the ETC complex I (Peruzzo et al. 2020).

In recent years, we have discovered that the activity of mitoBK_Ca_ channels in glioma cells is modulated by ETC substrates and inhibitors (Bednarczyk et al. 2013). This research proposed that cytochrome c oxidase plays a key role in regulating these channels. Furthermore, given that the activity of cytochrome c oxidase can be influenced by specific near-infrared (NIR) light wavelengths (Sanderson et al. 2018), it opens up intriguing new possibilities for the light regulation of mitoK channels (Szewczyk and Bednarczyk 2018).

Further research is essential to fully elucidate the functional implications of these interactions, which may represent a regulatory mechanism specific to mitoK channels. Although their precise nature and consequences remain incompletely understood, the coupling between the energy-producing system (electron transport chain) and the energy-dissipating system (mitochondrial potassium channels) may constitute a novel and intriguing form of mitochondrial regulation.

## Mitochondrial proteins neighborhood

The mitochondrion, as a semi-autonomous organelle, creates a distinct environment that, due to its unique role and properties, influences the activity of mitoK channels in a manner different from that of the plasma membrane environment, even when the potassium channels share the same structure. Therefore, due to the specific environment present in mitochondria, mitoK channels may be regulated in a specific manner. Knowledge of the molecular identity of some mitoK channels has enabled studies to identify their protein interaction partners. Examples include the mitoBK_Ca_ channel, whose protein is encoded by the KCNMA1 gene (Singh et al. 2013; Kulawiak et al. 2023), and the ROMK2 protein, a putative component of the mitoK_ATP_ channel (Foster et al. 2012; Laskowski et al. 2019).

Studies on the mitochondrial potassium channel ROMK2 have shown that it interacts with two lipid kinases: acylglycerol kinase (AGK) and diacylglycerol kinase ε (DGKE) located in mitochondria. Additionally, it was shown that the products of AGK and DGKE, lysophosphatidic acid (LPA) and phosphatidic acid (PA), increased the activity of ROMK2 channels reconstructed into planar lipid bilayers (Krajewska et al. 2024).

More detailed analyses of mitoBK_Ca_ channel interactions have been found in the mitochondria of brain and heart cells (Singh et al. 2016; Zhang et al. 2017). These include electron transport chain components and enzymes involved in mitochondrial metabolism, including the Krebs cycle. This confirms and strengthens the hypothesis regarding the regulation of the mitoBK_Ca_ channels by the activity of the ETC. On the other hand, direct interactions of the mitoK channels with electron transport chain complexes are not evident, as indicated by recent studies performed on glioblastoma multiforme cells (Kulawiak et al. 2023).

Of particular note is the interaction between the mitoBK_Ca_ channel and the HSP60 protein, which is likely responsible for channel targeting to mitochondria. Identifying this protein as a binding partner may bring us closer to understanding the mechanism by which the mitoBK_Ca_ channel targets its destination (Singh et al. 2016). Moreover, the demonstration of an interaction between the α subunit of the mitoBK_Ca_ channel and the outer membrane translocase receptor protein Tom22 in the study by Zhang and colleagues (Zhang et al. 2017). It has been shown that in the brain cells, combination HSP60 with the channel’s forming proteins may help explain the mechanism of import of these proteins into mitochondria via mitochondrial translocases.

In turn, studies of the mitoBK_Ca_ channel interaction in heart cells have shown that this channel interacts with the ADP/ATP translocase (ANT) located in the inner mitochondrial membrane (Zhang et al. 2017). Analysis of the results indicated that the mitoBK_Ca_ channel interaction occurs through the interaction of the transmembrane domains of the channel (Zhang et al. 2017). The discovery of the mitoBK_Ca_-ANT interaction raises questions regarding its physiological role, as well as their mutual influence on the function of each protein.

The structural–functional interactions of mitoK channels with other mitochondrial proteins suggest a new dimension in the regulation of these proteins. The identified protein–protein interactions suggest the existence of mitochondria-specific mechanisms regulating mitoK channels’ localization and activity, raising new questions regarding the role of proximal proteins in mitoK channels’ function.

## Mitochondria and protein kinases

One of the key post-translational modifications responsible for transmitting information about changes in the extracellular environment and propagating signals throughout the cell is protein phosphorylation. This modification regulates the structure, activity, transport, and interactions of specific proteins within signaling pathways. Mitochondrial proteins are no exception, as these organelles also possess a specific set of kinases and phosphatases that modulate their activity and interactions. Kinases involved in mitochondrial protein phosphorylation play a crucial role in regulating mitochondrial morphology, biogenesis, and function—processes essential for adaptation to dynamic cellular and environmental conditions (Budas and Mochly-Rosen 2007; Kotrasová et al. 2021). Depending on the stimulus, different sets of kinases, both mitochondrial and those occasionally translocated into mitochondria, are activated, which, through phosphorylation, modulate the activity of proteins located in specific mitochondrial compartments. Most protein kinases do not have a dedicated mitochondrial localization. However, there is increasing evidence of occasional and temporal translocation of protein kinases into mitochondria as a result of activation of various cellular signaling pathways (Kowalczyk et al. 2012, 2009; Kohda and Gemba 2005; Comyn et al. 2024; Krupska et al. 2017; Farid et al. 2025). These kinases modulate mitochondrial metabolism under stress conditions and may play a role, e.g., in protecting cells from death, e.g., by influencing ROS and ATP production.

Knowledge of the mitochondrial substrates of kinases and phosphatases is limited, as is our understanding of their transport pathways. Consequently, the highly challenging studies of mitoK channel’s protein phosphorylation remain only partially validated and often rely on indirect evidence. However, some studies provide clear evidence for the involvement of protein kinases in the modulation of mitochondrial channels activity (Hawrysh et al. 2016).

Among mitochondrial proteins, the mitoK channels are also regulated by specific kinase interactions through specific phosphorylation. In isolated cardiac mitochondria, activation of protein kinase C (PKC) during ischemia has been shown to protect mitochondria from damage through the activation of mitoK_ATP_ channels (Korge et al. 2002). Studies on enriched mitochondrial fractions from turtle brain demonstrated phosphorylation of the putative mitoK_ATP_ channel subunit Kir6.2 at Thr-224, with phosphorylation levels increasing under hypoxic conditions. This effect was abolished by the kinase inhibitor staurosporine. However, these studies did not identify the kinase responsible for this modification, and the involvement of PKCε in this process was excluded. Furthermore, this modification does not appear to contribute to hypoxia-induced depolarization of the mitochondrial membrane potential (ΔΨ) (Hawrysh et al. 2016). A role for PKCε and mitoK_ATP_ channels in protecting cardiomyocytes from ischemia has been demonstrated in vitro (Liu et al. 2002). Similarly, studies of Ca^2+^-induced injury in rat myocardium suggest that the protective effect of the mitoK_ATP_ channel against Ca^2+^ overload is mediated by PKC-related signaling pathways, in which PKCδ was observed to translocate into cardiomyocyte mitochondria under the pretreatment with diazoxide, opener of mitoK_ATP_ channel (Wang and Ashraf 1999; Takashi et al. 1999). Additional studies employing diverse pharmacological approaches in models of cardiac ischemia demonstrated that the cardioprotective effect of mitoK_ATP_ channels involves the translocation of phosphorylated Akt kinase from the cytosol to the mitochondria (Ahmad et al. 2006). Furthermore, experiments examining potassium flux in liposomes containing a partially purified mitoK_ATP_ channel showed that mitochondrially located PKCε and the mitoK_ATP_ channel can associate in a stable and functional manner (Jaburek et al. 2006). Finally, evidence from studies on human myocardium indicates that mitoK_ATP_ channel opening, PKC activation, and p38MAPK activation represent essential steps in the signal transduction cascade underlying ischemic cardioprotection and pharmacological preconditioning. In this pathway, PKC activation appears to occur downstream of mitoK_ATP_ channel opening and upstream of p38MAPK activation (Loubani and Galiñanes 2002).

Studies in rabbit hearts demonstrated that inhibition of phosphodiesterase 5 (PDE5) by sildenafil induced cardiomyocyte protection in a manner sensitive to the mitoK_ATP_ channel blocker 5-hydroxydecanoic acid (5-HD) (Ockaili et al. 2002). Similarly, protection conferred by sildenafil and by the alternative PDE5 inhibitor tadalafil in perfused mouse hearts was abolished by KT5823, the cGMP-dependent protein kinase I (cGKI) inhibitor, suggesting that signaling from cGMP to mitoK_ATP_ is mediated by this kinase (Das et al. 2008; Salloum et al. 2009). Nevertheless, caution is warranted when interpreting the relationship between PDE5, PDE5/cGMP inhibition, and the function of mitoK channels in cardiomyocytes during ischemia–reperfusion, as the presence of PDE5 in adult mouse cardiomyocytes remains questionable. Moreover, the protective effect of sildenafil may arise, at least in part, from vasodilatory mechanisms rather than direct modulation of mitochondrial channels (Ockaili et al. 2002; Lukowski et al. 2022).

Taking into account the interactions of mitoK channels with ETC subunits, modulation of their activity through phosphorylation may influence the channel state (open/closed). Phosphorylation by PKA, PINK1, CDK1, and SRC kinases increases the activity of complex I (NADH dehydrogenase). Succinate dehydrogenase (complex II) is regulated by the action of FGR and SRC kinases. Cytochrome c oxidase (complex III) activity is upregulated by phosphorylation via SRC kinase. Cytochrome c activity is impaired after phosphorylation by AKT and AMPK. Complex IV (cytochrome c reductase) is negatively regulated by PKA (independently of cAMP levels) and positively regulated by cAMP-dependent PKA and PKCε activity (Acin-Perez et al. 2010; Kotrasová et al. 2021). Furthermore, PKCδ inhibits ATP synthase activity through interaction with the “d” subunit of F_1_F_O_-ATP (Nguyen et al. 2008).

The mitoK channels appear to be tightly regulated by kinases, particularly PKC, Src, and AMPK. This regulation influences mitochondrial function under physiological and pathological conditions (Frankenreiter et al. 2017). A better understanding of these interactions offers hope for effective treatment of diseases associated with mitochondrial dysfunction, such as cardiac ischemia, neurodegeneration, and cancer.

## Metabolic regulation of mitochondrial potassium channels

It is undeniable that mitochondria are central organelles in cellular metabolism and function as hubs integrating both metabolic and signaling pathways (Picard and Shirihai 2022). The presence of mitoK channels with diverse biophysical properties, conductance characteristics, and environmental sensitivities allows them to respond specifically to numerous signaling pathways as well as to factors influencing mitochondrial function and metabolism (Rotko et al. 2020a, b). Mitochondrial metabolic activity is tightly coupled to the cell’s demand for ATP, fluctuations in calcium ions concentration and its transport across the IMM, and the synthesis of heme, an essential cofactor for the ETC proteins. Alterations in ETC activity result in differential ROS production, which—depending on concentration—may act as signaling molecules or trigger cells apoptosis and necrosis (Lewandowska et al. 2024). These factors, in turn, directly influence the activity of mitoK channels, some of which play critical roles in mitochondrial regulation. A few examples of such regulatory interactions merit particular attention.

One group of mitoK channels comprises those regulated by Ca^2+^, namely mitoBK_Ca_, mitoIK_Ca_, and mitoSK_Ca_, which differ in conductance (Siemen et al. 1999; De Marchi et al. 2009; Dolga et al. 2013; Stowe et al. 2013). The coupling between their activity and mitochondrial matrix Ca^2^⁺ concentration is crucial, given the role of calcium in regulating enzymes of the Krebs cycle. Since the activity of these enzymes depends on the availability of respiratory substrates that fuel the ETC, Ca^2^⁺ influx into the mitochondrial matrix plays a dual role: on one hand, it stimulates Krebs cycle enzymes, but on the other, it activates Ca2⁺-regulated mitoK channels (mitoBK_Ca_, mitoIK_Ca_, or mitoSK_Ca_). Activation of these channels decreases the IMM ΔΨ, thereby limiting further Ca^2^⁺ influx into the matrix and reducing the activity of Krebs cycle enzymes (Siemen et al. 1999; De Marchi et al. 2009). The Ca^2^⁺-activated mitoK channels are expressed in various tissues and cell types, underscoring their importance in the regulation of mitochondrial metabolism (Augustynek et al. 2017; Trombetta-Lima et al. 2020). By modulating Ca^2^⁺ influx through the inner mitochondrial membrane, these channels may constitute an important element of cytoprotective mechanisms. In particular, controlling excessive Ca^2^⁺ entry helps prevent overactivation of mitoIK_Ca_ channels, which are triggered under conditions of markedly elevated Ca^2^⁺ concentrations in the mitochondrial matrix (Xu et al. 2002; Stowe et al. 2013).

Another key mitochondrial metabolite is ATP, generated through oxidative phosphorylation. Fluctuations in ATP levels can directly influence the activity of mitoK_ATP_ channels, as these channels are inhibited by ATP within the matrix. A decline in ATP concentration likely leads to channel activation, which in turn causes depolarization of the inner mitochondrial membrane and a subsequent increase in electron transport chain activity.

Despite substantial evidence supporting the existence and activity of mitoK_ATP_ channels in mitochondria from various tissues and cell types, their precise molecular structure still remains a matter of debate, giving rise to several hypotheses. For instance, current research suggests that the mitoK_ATP_ channel may be formed by the mitoK protein (CCDC51) together with the mitoSUR receptor (Paggio et al. 2019). Other studies, however, propose that it may consist of ATP synthase subunits or a ROMK-type channel (Foster et al. 2012; Laskowski et al. 2019). Regardless of these controversies, the presence of an ATP-sensitive mitochondrial channel is well documented, and it is likely to represent a key component of signaling pathways that respond to changes in mitochondrial metabolism.

In addition to the factors mentioned above, heme is an important mitochondrial component that regulates the activity of certain mitoK channels. Heme functions not only as a cofactor for numerous enzymes, including mitochondrial ETC complexes, but also as a redox sensor and signaling molecule, thereby linking mitochondrial function to broader cellular metabolic states. The mitoBK_Ca_ channels are modulated by heme and its oxidized form, hemin (Augustynek et al. 2014; Rotko et al. 2020a, b; Walewska et al. 2022). Recent studies have demonstrated that both heme and hemin can inhibit mitoBK_Ca_ channel activity, most likely through binding to the heme-binding motif (CXXCH) located between the two RCK domains of the channel’s α subunit. Moreover, heme binding appears to be essential for carbon monoxide (CO)-dependent regulation of mitoBK_Ca_ channels, as CO has little effect on channel activity in the absence of heme (Rotko et al. 2020a, b). Together, these findings underscore the complex regulation of mitoBK_Ca_ channels by heme and hemin, highlighting their role in linking mitochondrial ion channel activity to redox states and intracellular signaling pathways.

In summary, recent studies clearly demonstrate that mitoK channels do not merely serve a passive role in K⁺ transport but also exert a significant influence on mitochondrial metabolism in response to changing cellular demands. Moreover, they actively contribute to cell protection, for example, under hypoxic conditions, and regulate not only mitochondrial but also overall cellular metabolism.

## Conclusions and perspectives

Over the past 34 years since the identification of the first potassium channel in the IMM, research in this field has made substantial progress, revealing that mitoK channels play important roles in intracellular signaling pathways (Kulawiak and Szewczyk 2022). The localization of mitoK channels within mitochondria—organelles that serve as central hubs for metabolism—points to novel and unique mechanisms of mitoK channels’ regulation that are only beginning to be understood (Szewczyk 2024). Several of these mechanisms have been discussed in this paper.

Nevertheless, other distinctive regulatory pathways of mitoK channels remain to be elucidated. Recently, we have shown the presence of mechanosensitive mitoBK_Ca_ channels in mitochondria of astrocytoma cells (Walewska et al. 2018). Our findings indicate the possible involvement of the mitoBK_Ca_ channels in mitochondria activities in which changes in membrane tension and shape play a crucial role, such as fusion/fission and cristae remodeling. Since mitochondria undergo dynamic structural changes, this kind of mitoK channels’ regulatory mechanism may be an important signaling element within the cell (Sek et al. 2025).

Moreover, the regulation of mitochondrial potassium channels by phospholipids has emerged as an important area of investigation (Pipatpolkai et al. 2021). Cardiolipin (CL), a mitochondria-specific phospholipid essential for maintaining mitochondrial structure and function, has been shown to modulate the behavior of some potassium channels (Fuentes and Morcillo 2024). As a negatively charged lipid, CL directly interacts with channels such as KcsA, enhancing their activity and promoting channel opening (Iwamoto et al. 2023). CL regulation of mitoK channels may open a new area of investigation concerning cristae remodeling.

Additionally, our studies have shown that the activity of mitoBK_Ca_ channels in glioblastoma cells is influenced by substrates and inhibitors of the mitochondrial ETC (Bednarczyk et al. 2013). These observations point to cytochrome c oxidase (COX) as a critical component in the regulation of mitoBK_Ca_ channels. Considering that COX is the main infrared-absorbing protein in mitochondria, the possibility of light-dependent regulation of mitoK channels has been proposed (Szewczyk and Bednarczyk 2018), opening an intriguing avenue for future research on non-pharmacological regulation of mitoK channels.

**Fig. 1:**
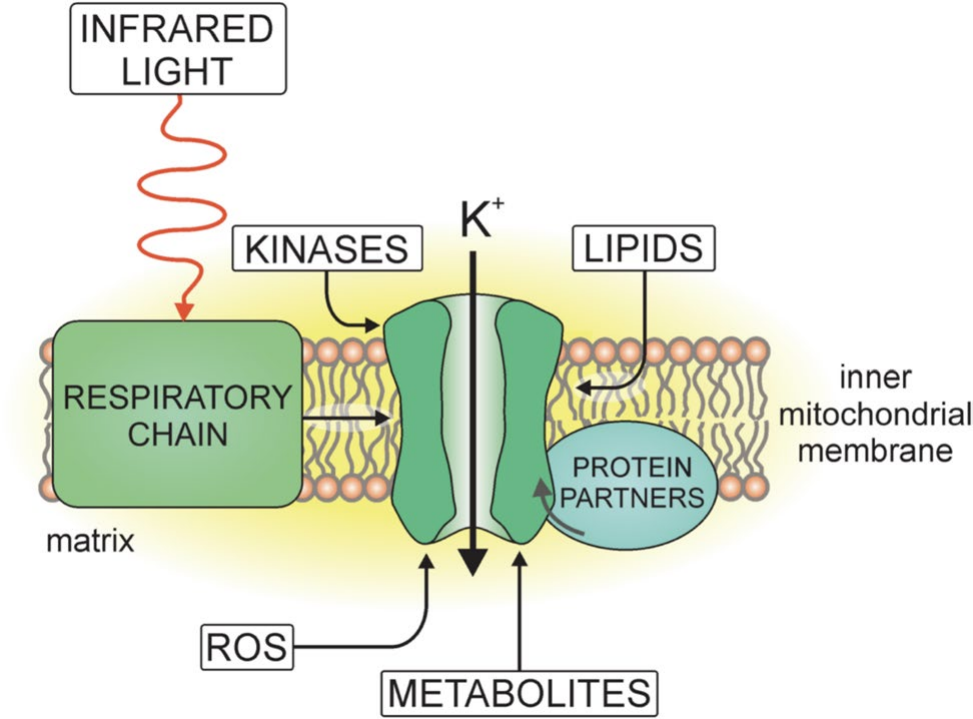
Regulation mechanisms targeting potassium channels present in the inner mitochondrial membrane and discussed the manuscript. ROS, reactive oxygen species

Looking ahead, the exploration of mitoK channels’ unique regulation mechanisms reveal complex and unexpected links between biophysics of ion transport, bioenergetics, and cell signaling (Fig. 1). Understanding these mechanisms in detail will not only deepen our knowledge of mitochondrial physiology but probably may also lead to new therapeutic strategies targeting diseases associated with mitochondrial dysfunction (Garlid 2000). Future efforts should focus on resolving the molecular identity of these channels, elucidating their mitochondrial regulatory mechanisms, and uncovering their roles in cellular function.

## Data Availability

Not applicable.
